# Negative Requests Within Hair Salons: Grammar and Embodiment in Action Formation

**DOI:** 10.3389/fpsyg.2021.689563

**Published:** 2022-07-15

**Authors:** Anne-Sylvie Horlacher

**Affiliations:** ^1^Center for Applied Linguistics, University of Neuchâtel, Neuchâtel, Switzerland; ^2^French Linguistics and Literary Studies, University of Basel, Basel, Switzerland

**Keywords:** negative requests, action formation, service encounters, hair salon interactions, expert-novice categories, conversation analysis, grammar-body interface, French

## Abstract

Although requests constitute a type of action that have been widely discussed within conversation analysis-oriented work, they have only recently begun to be explored in relation to the situated and multimodal dimensions in which they occur. The contribution of this paper resides in the integration of bodily-visual conduct (gaze and facial expression, gesture and locomotion, object manipulation) into a more grammatical account of requesting. Drawing on video recordings collected in two different hair salons located in the French-speaking part of Switzerland and in France (23 h in total), this paper analyzes clients’ negative requests by exploring how they interface with the participants’ embodied conducts. Contrary to what the literature describes for positively formulated requests, with negative requests clients challenge an expectable next action (or ongoing action) by the hairdresser. One linguistic format constitutes the focus of this article, roughly glossable as ‘You don’t do [action X] too much (huh)’. Our analysis of a consistent collection of such formatted turns will show that clients present them (and hairdressers tend to treat them) in different ways, depending on how they relate to embodied conduct: When these turns are used by the client as instructions, they are accompanied by manipulations of the client’s own hair and tend to occur toward the initial phase of the encounter, at a stage when hairdressers and clients collaboratively negotiate the service in prospect. When uttered as directives, these turns are not accompanied by any touching practices from the client and are typically observable in subsequent phases of the encounter, making relevant an immediate linguistic or/and bodily response from the professional, as shown by the client who is actively pursuing mutual gaze with him/her. Therefore, an action cannot be distinguished from another on the basis of the turn format alone: Its sequential placement and the participants’ co-occurring embodied conduct contribute to its situated and shared understanding. By analyzing the clients’ use of a specific linguistic format conjointly with the deployment of specific embodied resources, this study will advance our understanding of how verbal resources and embodiment operate in concert with each other in the formation and understanding of actions, thereby feeding into new areas of research on the grammar-body interface.

## Introduction

Research on requests is prolific within conversation analysis-oriented work, as evidenced in several recent publications, among which most notably Drew and Couper-Kuhlen’s (2014) volume *Requesting in Social Interaction* (but see also Curl and Drew, 2008; Rossi, 2015; Kendrick and Drew, 2016; Zinken, 2016).^1^ Although requests constitute a type of action that have been widely discussed in the literature, they have only recently begun to be explored in relation to the speakers’ co-occurring embodied conducts (Fox and Heinemann, 2016; Mondada, 2018, 2019). These aspects are here tackled in relation to a specific environment—the hair salon—in which the professional service deals with the clients’ physical appearance, thereby involving the participants’ bodies in crucial ways.

Drawing on multimodal conversation analysis (Streeck et al., 2011) and interactional linguistics (Selting and Couper-Kuhlen, 2001; Couper-Kuhlen and Selting, 2018), this paper investigates moments in which a hairdresser’s embodied (future) action is challenged by the client, who self-selects and displays a certain degree of authority over how her/his hair should be treated, cut, or styled: *(vous) coupez quand même pas tro:p hein* ‘(you) don’t cut too much huh’/*vous faites pas trop de p’tites boucles hein* ‘you don’t make too many little curls huh,’ etc. While the grammatical features of these turns are strikingly similar, clients may carry out different kinds of actions with these turns, which can be presented (and interpreted) as instructions (i.e., later requests, being relevant for the future treatment), or directives (i.e., urgent requests, calling for immediate compliance).^2^ The analysis suggests that clients display by their co-occurring embodied conducts what type of action they are accomplishing with these turns. As will be shown later, we can define and recognize these different social actions by considering the embodied formation of the client’s action, but also the timing of the client’s action with relation to the ongoing activity (consultation phase vs. hair-treatment phase). The analysis will also reveal that the hairdressers respond to an instruction by displaying their compliance, whereas they respond to a directive by modifying their ongoing embodied action. The research questions stemming from this study are thus as follows: What new insights can be gained when investigating a specific grammatical format in relation to the speakers’ co-occurring bodily-visual conducts? How do the recipients come to understand these combinations as implementing specific actions?

This paper contributes to four domains: (1) It sheds light on the complex interplay between grammar and embodied conduct in action formation; (2) it contributes to current research on request formats and issues of expertise and entitlement, by analyzing a setting in which delicate negotiations regarding the clients’ tastes and personal preferences are at stake; (3) it reflects current concerns with respect to institutional talk and its specific organization, by adding to our understanding of what ‘servicing’ the clients—with its constraints and complexities—means in this setting of interaction; and (4) it focuses on French and thus brings variety into the field of interactional linguistics, which is still dominated by research on English.

## Requests

Requests are basic and crucial actions in social interaction. For the purpose of this study, a request is defined as a social action, accomplished by means of a set of linguistic and embodied resources, by which a participant solicits someone to do something (e.g., providing a service, information, help, etc.). Requests are *first actions* that project compliance as *seconds* (= the requested action; Mondada, 2014).

Section ‘Requests’ is organized as follows: section ‘Requests and Other Actions: Action Formation and Ascription’ discusses requests along with other first actions, providing some insights on action formation and ascription in social interaction. Section ‘Requests in (Hairdressing) Service Encounters’ reviews works that have dealt with requests in service encounters, including studies focusing on requests in hair salons, showing that, in this setting, the professional service deals with the clients’ bodies, and most specifically, their heads (see section ‘Touching the Body’). This aspect is consequential for how asymmetries and institutional categories are negotiated on the basis of specific rights and obligations (see section ‘Expert-Novice Categories’).

### Requests and Other Actions: Action Formation and Ascription

Research in conversation analysis and interactional linguistics has discussed requests along with other first actions: *instructions* (Deppermann, 2015, 2018), *blames/complaints* (Schegloff, 2005; Heinemann and Traverso, 2009), *orders/directives* (Goodwin, 2006; Cekaite, 2010; Craven and Potter, 2010; Mondada, 2011, 2013b,^2017^), *critiques* (Mondada, 2013a, b), etc. Schegloff (2007, p. xiv) presents *the action formation problem* in this way: “How are the resources of the language, the body, the environment of the interaction, and position in the interaction fashioned into conformations designed to be recognized by recipients as particular actions?” If language is an important resource, grammar alone is not sufficient for action recognition (Kent and Kendrick, 2016). A directive may be realized by a great number of different grammatical formats, and namely, a declarative (Skogmyr Marian, 2018). A request may take the form of a statement of need or desire (Vinkhuyzen and Szymanski, 2005; Rønneberg and Svennevig, 2010). Social actions thus cannot be distinguished in terms of their lexical, morphosyntactic, and prosodic patterning. In addition, some utterances may allow implementing two actions at the same time, one action serving as a vehicle for another “primary” (Levinson, 2013, p. 127) action (on the double-barreled nature of actions, see also Schegloff, 2007, p. 76; Rossi, 2018). Again, this raises the much-debated question of how words relate to action (Levinson, 2013; Couper-Kuhlen, 2014; Sidnell, 2017; Enfield and Sidnell, 2017).

In talk-in-interaction, it is through second actions that participants display their understanding of a preceding first action, for instance as a request. While conversation analysis uses the *next-turn proof procedure* (Sacks et al., 1974), relying on the recipient’s next action is “not always a source of unequivocal validation” (Heritage, 2012, p. 80; on action ascription, see also Levinson, 2013; Deppermann and Haugh, 2022). In addition, Heritage (2012) introduces *epistemic status* as an unavoidable component of the production and recognition of social actions (see, however, Lindwall et al., 2016 for a discussion). In this view, turns-at-talk formatted with modal verbs may still be recognized as directives projecting unquestionable compliance if uttered by a highly entitled participant. Reversely, turns-at-talk formatted with imperatives may be interpreted as proposals keeping compliance negotiable to some extent if uttered by a less entitled speaker (on grammatical format and entitlement, see also Heinemann, 2006; Craven and Potter, 2010; Antaki and Kent, 2012).

More recently, the focus on the body in research on social interaction (Nevile, 2015) has shown that embodied aspects of human conduct are consequential for the formation and interpretation of actions (see Kärkkäinen and Keisanen, 2012 for offers, Skogmyr Marian, 2021 for complaints, Sorjonen and Raevaara, 2014 for requests). However, previous research has not shown the ways in which the same grammatical format may deliver different interpretations to the type of action being implemented, depending on the speakers’ distinctive co-occurring embodied conducts. By exploring how a grammatical format interfaces with the participants’ embodied conducts in beauty care encounters, this study hopes to make a significant step forward in understanding how verbal resources and embodiment work in concert with each other in the realization and interpretation of specific actions (requests, but also instructions, directives, etc.), thereby feeding into new areas of research on the grammar-body interface (Pekarek Doehler, 2016, 2019; Couper-Kuhlen, 2018; Keevallik, 2018; Streeck, 2018; Maschler et al., 2020).

### Requests in (Hairdressing) Service Encounters

Requests are ubiquitous in previous research on service encounters (Merritt, 1976; Aston, 1988; Félix-Brasdefer, 2015). They are documented in settings such as convenience stores (Sorjonen and Raevaara, 2014; Mondada and Sorjonen, 2016), cheese shops (Mondada, 2018), shoe repair shops (Fox and Heinemann, 2015, 2016, 2021), beauty supply stores (Ryoo, 2005), restaurants (Kuroshima, 2010), public bars and coffeehouses (Richardson and Stokoe, 2014; De Stefani, 2019), and bookstores (Aston, 1988; Mansfield, 1988). Requests have also been investigated in other settings, such as public employment services (Asmuß, 2007), train station counters (Hausendorf and Mondada, 2017), theater box offices (Lindström et al., 2017), university help desks (Mortensen and Hazel, 2014), and service phone calls, ranging from emergency calls (Wahlen and Zimmerman, 1990; Fele, 2006; Drew and Walker, 2010) to advice and mediation helplines (Emmison and Firth, 2012; Sikveland and Stokoe, 2016). In all these settings, request formats (e.g., *need/want-declaratives, can-interrogatives, wonder-clauses*, Fox and Heinemann, 2016)—sometimes composed by a unique word (e.g., the name of a product or a demonstrative with a pointing gesture, Mondada, 2019) may vary with respect to the nature of the service that is requested, the grantability of the request, the requester’s degree of entitlement to make the request, and so on (see Fox and Heinemann, 2016 for a summary; see also Lindström, 2005; Zinken and Ogiermann, 2011; Fox, 2015). However, previous work has mainly studied settings in which the service consists in buying a product (object transaction) or in seeking assistance, information, or advice (counseling). Yet another part of our everyday service encounters involves clients’ physical and mental well-being, such as hairdressing (Oshima, 2009, 2014, 2018; Greer, 2013; Oshima and Streeck, 2015; Nizameddin, 2016; Heinrichsmeier, 2020), fitness training (Bolin, 1992; Gimlin, 2002), facial care (Toerien and Kitzinger, 2007a,b), massage, relaxation (Nishizaka and Sunaga, 2015), manicure (Nizameddin, 2016), and others. Despite the social and financial impact of these services, beauty and wellness treatment interactions remain largely understudied. This paper contributes to filling this gap in current research by analyzing requests in the interaction between service providers and clients in hairdressing service encounters.

A large amount of the clients’ requests in hairdressing service encounters concern issues related to revision (see pioneering work by Oshima, 2009). Clients are expected to trust their professionals but at the same time they are co-responsible for the outcome of the service. The ways in which clients identify potential problems located on their heads and how hairdressers achieve the identification of the different problematic areas/objects that clients make visible to them is not a straightforward issue (Horlacher and De Stefani, 2017). Clients do not always explain verbally what a problem is with their hair. The professionals might already interpret the clients’ touching their own hair and displacing some strands as a request for revision. This suggests that requests in hair salons need to be identified also on the basis of recurrent, embodied conduct (see section ‘Introduction’).

Other studies on hairstyling have shown that during episodes of chat (on *small* talk, see e.g., Holmes, 2000), the client may initiate a concurrent, task-related first action, typically by producing a request, thereby prioritizing the professional activity at hand (De Stefani and Horlacher, 2018; Horlacher, forthcoming). The hairdresser complies with the client’s request, after which the interactants resume chatting. The hairdressers’ work includes managing the dual demands of conversational talk and professional activity (Heinrichsmeier, 2020). The ways in which practitioners adjust their professional practices in order to face contingent and unplanned situations lies at the core of the service they offer to their clients (LeBaron and Jones, 2002; Nizameddin, 2016).

Negative requests have come up tangentially in Horlacher (2017), in which we investigate the lexico-syntactic formats of the clients’ requests and show how they may be related to the participants’ rights and obligations. Clients can be seen to challenge the hairdressers’ taken-for-granted authority, as well as their professional expertise and social identity through negative requests. Other studies conducted in different settings have analyzed question-like formats as challenges (Heritage, 2002; Koshik, 2003). To give but one example, Monzoni (2009) analyzes negatively framed questions with declarative syntax in institutional calls to the ambulance emergency service, arguing that the negative format is challenging in Italian. In her data, negative-formatted turns (such as *i pazienti, oggi domani non li andate a prendere?* ‘the patients, today tomorrow you don’t go and pick them up?’) are produced as direct complaints (but see also Heinemann, 2006 for another highly relevant study on ‘negative interrogative requests’ and issues of entitlement).

In this paper about hairdressing, we further expand this line of interest about negative-formatted turns by focusing on one singular format used by the clients (‘You don’t do [action X] too much (huh)’). We show how this specific linguistic format may be related to the clients’ ‘expertise’ and entitlement, and we analyze the recurrent combination of this format with an observable embodied conduct.

#### Touching the Body

In beauty treatment encounters, the professional service deals with the clients’ physical appearance. Requests in this environment thus involve the bodies of the participants in significant ways, just as requests occurring during tattoo sessions (Nizameddin, 2016) or encounters between clients and photographers (Tekin, 2017; Mondada and Tekin, 2020). The client requests a specific service through different linguistic and embodied practices, while granting the request requires the professional to delicately touch and manipulate the client’s hair and the head.^3^ Hence, requesting in this setting most often involves a fairly *intimate dimension* (Cekaite and Mondada, 2020), even though participants tend to treat haptic contacts as a manifestation of *professional touch* (Mondada and Tekin, 2020). If, for the professional, the client’s body is ‘objectified’ as a working space on which he/she performs technical tasks, for the client, the professional’s interventions on these body-parts goes hand in hand with negotiations of requests, decisions, and entitlements. Moreover, the service deals with *irreversible* (Horlacher, 2017) body modifications such as hair cutting, coloring, and removal. The ways in which the hairdressers understand the clients’ demands are crucial for a successful service.

#### Expert-Novice Categories

In hairdressing service encounters, participants display their *membership categories*, which accord them specific rights and obligations (Sacks, 1984, 1992). Typically, if clients visit hair salons, they assume hairdressers to be competent in satisfying their demands (see Tekin, 2017 for a related argument in encounters between clients and photographers). However, the client’s initial request is progressively shaped and transformed in accordance with the beauty specialist’s expertise and professional vision (Goodwin, 1994). On the one hand, clients are entitled to their own opinions about their appearance. On the other hand, professionals are expected to listen to the clients’ desires about the outcome of the service, but have the responsibility to tell them whether their requests can be granted or not (on non-granting requests in commercial service encounters, see also Lee, 2011). Clients can resist the advice given by professionals and initiate competing actions, thereby reversing the participants’ asymmetric relations (Mondada and Keel, 2017; Tekin, 2017). Hence, we can hardly categorize clients in beauty treatment encounters as ‘non-experts,’ given that “they endorse expert stances that may contradict their normative role expectations as service recipients” (Jacobs-Huey, 1996, p. 47). Likewise, professionals must juggle their status of beauty experts and service providers (Oshima, 2009), while aligning with the client’s concerns. In sum, in the service encounters analyzed here, the clients entrust their bodies and heads to a professional, while at the same time claiming authority over their bodies and remaining entitled to their own opinions about their appearance. All these aspects highlight the specificity of the ‘You don’t do [action X] too much (huh)’ format as it is used by the clients in our data.

## Data, Methods, and Sketch of the Analysis

The data on which this article is based consist of 23 h of video recordings collected in 2010, 2013, and 2018 in two different hair salons located in the French-speaking part of Switzerland and in France. Both hair salons in which the recordings took place are local businesses run by male owners who are themselves working in the salon, while supervising a team of three to four other certified hairdressers. The excerpts selected for this paper show interactions taking place between the two owners of their respective salons and four different regular male and female clients (ex. 3 and ex. 4 involve the same client).

Working on our hairdressing data, we came across 10 negative-formatted turns through which clients express what they do not want instead of what they do want. In the 10 excerpts identified in our corpus, the clients orient toward a possible negative outcome of the hairdressers’ ongoing or projected action. One might argue that the ‘you don’t do [action X] too much (huh)’ seems to be quite a rare practice if it occurs every 2 h or so. However, the data at our disposal consist of 13 sessions lasting 1–2 h each, involving 13 clients. Therefore, these numbers suggest that almost every client (CLI) makes one negative request by mobilizing this format during her or his encounter with the hairdresser (HAI). Taking this particular request format as a starting point, we selected five excerpts and decided to investigate CLIs’ negative-formatted turns in accordance with four relevant dimensions: (a) *grammar*, by looking at the linguistic (morpho-syntactic, lexical) resources that CLIs use when formatting their turns; (b) *temporality*, by paying attention to when these turns occur with regard to temporal contingencies; (c) *sequentiality*, by analyzing how HAIs respond to CLIs’ turns; and (d) *bodily-visual conduct*, by investigating the participants’ co-occurring embodied conducts when these turns are uttered. The analysis will show that by using similar verbal turns accompanied by different embodied resources in different interactional contexts, the participants accomplish different social actions.

The study combines methods of conversation analysis (Sacks et al., 1974), interactional linguistics (Ochs et al., 1996; Hakulinen and Selting, 2005; Couper-Kuhlen and Selting, 2018), and the analysis of embodied interaction (Streeck et al., 2011). Working toward a holistic understanding of language, these methods have been undertaken to analyze grammar as inextricably intertwined with other semiotic resources such as gesture, gaze, body posture, and object manipulation. More importantly, the analysis aims to show the ways in which embodied conduct plays into such fundamental issues as action formation and ascription. In doing so, the approach seeks to identify *multimodal action packages* (Goodwin, 2007), i.e., recurrent co-occurring constellations of verbal-prosodic-embodied features (Pekarek Doehler, 2019) that allow for a better understanding of the interplay between grammar and embodiment in the processes whereby participants accomplish, coordinate, and interpret their respective actions.

In the remainder of section ‘Data, Methods, and Sketch of the Analysis,’ we briefly comment on the lexico-syntactic features of the clients’ turns (see section ‘Grammatical Features of Clients’ Requests’) and their temporality (see section ‘Sequential Environments of Clients’ Requests’). The other dimensions will be thoroughly discussed in the analysis (see section ‘Analysis’).

### Grammatical Features of Clients’ Requests

The five negative-formatted turns that we are interested in are reproduced below. CLIs each point to an action that may have irrevocable consequences. All these turns are treated by HAIs as being prescriptive but have a declarative syntax (see, however, ex. 4). In Excerpt 4, indeed, the initial *(vous)* ‘(you)’ is hearable only very tentatively. If it is not there, CLI’s turn would be more recognizable as an imperative. Interestingly, almost all turns (except ex. 1) are formatted with turn-final *hein*, which may be described as an *interrogative particle* in French (Mondada, 2013a)^4^ :

**Excerpt 1**


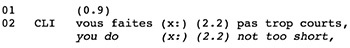


**Excerpt 2**


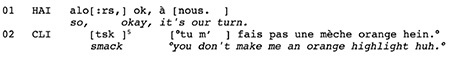


**Excerpt 3**


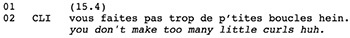


**Excerpt 4**


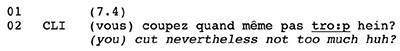


**Excerpt 5**


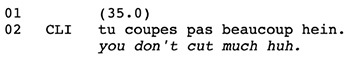


The CLIs’ turns are built:

• as a negative declarative (or imperative; see ex. 4)
• in present tense^6^
• with second person singular (*tu* ‘you’) or plural (polite) form (*vous* ‘you’) as subjects of the clause
• including verbs like ‘to cut’ or ‘to do’ (i.e., relating to possibly irreversible actions)
• including a quantifier associated to the verb (but see ex. 2)
• with *huh* as a turn-closing device (but see ex. 1)

CLIs’ negative requests emerge as first actions, sometimes after a silence of more than 30 s. The second action in these turns consists for the professionals not in doing something but in *not* doing something (about negative directives, see Mondada, 2011, 2013b; about negative declaratives, see Keevallik, 2009; Monzoni, 2009; Seuren and Huiskes, 2017). In these encounters, however, negative requests are sometimes formulated *in response* to the hairdressing activity (ex. 3, 4, and 5). In that sense, it might be peculiar to think of first actions as responding to something. A sequential analysis will show how, as first actions, these negative requests are embedded into the wider contextual circumstances in which they occur, being finely tuned to the temporal unfolding of the hairdressing activity.

In what concerns the negative form, in all excerpts CLIs face a ‘risky’ situation: They are expecting an action from HAIs which they interpret as negative. CLIs’ use of the negation does not primarily relate to preceding talk (but see ex. 1), as the nominated actions have not been negotiated in the prior discussion. This suggests again that the negative formulation seems to be linked to what is happening (or projected) in the hairdressing activity at that moment, more than to what the participants have been talking about in their prior turns. In some cases (ex. 1, 2, 5), however, it will be difficult to claim that CLIs’ negative requests take into account possible misunderstandings or problems arising in the hairdressing activity, as the professional has not yet initiated the action to which CLIs orient at that moment. Therefore, CLIs’ negative requests may also rely on the participants’ past history, previous services, common beliefs, and shared assumptions^7^ (see Deppermann and De Stefani, 2019 on the role of negation in definition activities).

### Sequential Environments of Clients’ Requests

Apart from their grammatical realization, another relevant aspect when describing CLIs’ turns is their occurrence with regard to temporal contingencies.

CLIs’ negative requests typically occur during the initial phase of the encounter (ex. 1 and 2) when HAIs and CLIs negotiate the service to be delivered. At this stage of the encounter, both participants are engaged in *the talking part* vs. *the doing part* (Lindwall et al., 2015) of the request, that is, the CLIs’ requests are not immediately turned into courses of actions. These negative requests can be described as instructions (Lindwall et al., 2015) whereby the clients make the professionals understand what they are supposed to do with their hair. Instructions are relevant for the whole treatment. Their aim is not just to make the hairdresser perform a certain action at a particular moment.

Occurrences of negative requests are also observable in subsequent phases of the encounter (ex. 3, 4, 5), in the midst of the procedure. At this stage, these negative requests can be described as directives (Craven and Potter, 2010), as they have to be complied with urgently (on later and now-directives, see Vine, 2009).

In both cases, CLIs have to evaluate when the point of no return is reached. All these turns are preventive in some sense, but the analysis will further define the urgency in the CLIs’ turns. In some excerpts, CLI self-selects well before the nominated action whereas, in others, the HAI’s action is imminent or already underway. The analysis will further identify the type of action CLIs’ turns are brought to accomplish in precise sequential environments. It will also look at how this specific turn format and embodied resources combine to constitute either instructions or directives.

## Analysis

In what follows, we show that when CLIs’ turns are later requests (see section ‘Later Requests and Self-Touching Practices’), they are typically accompanied by manipulations of CLI’s own hair. These later requests can be described as instructions (ex. 1), sometimes tainted with criticism (ex. 2). By contrast, when CLIs’ requests require immediate action (see section ‘Urgent Requests and Gaze Search’), they can be described as directives and are not associated with any touching practices from CLI. What we observe is that CLI is closely monitoring HAI’s activity, and actively pursuing mutual gaze with the professional, thereby seeking compliance with the action that is called for (ex. 3, 4, and 5). Therefore, the ways in which CLIs’ embodied conducts may affect action formation and ascription are issues that are most relevant to address in this context.

### Later Requests and Self-Touching Practices

Excerpt 1 occurs toward the initial phase of the encounter. CLI makes the professional understand---both verbally and manually---how much he is supposed to cut. The consultation phase is not the only opportunity that CLI has in negotiating the service but it is the first moment in which he or she gives instructions to the professional, like in the following excerpt^8^ :

**EXCERPT 1:**
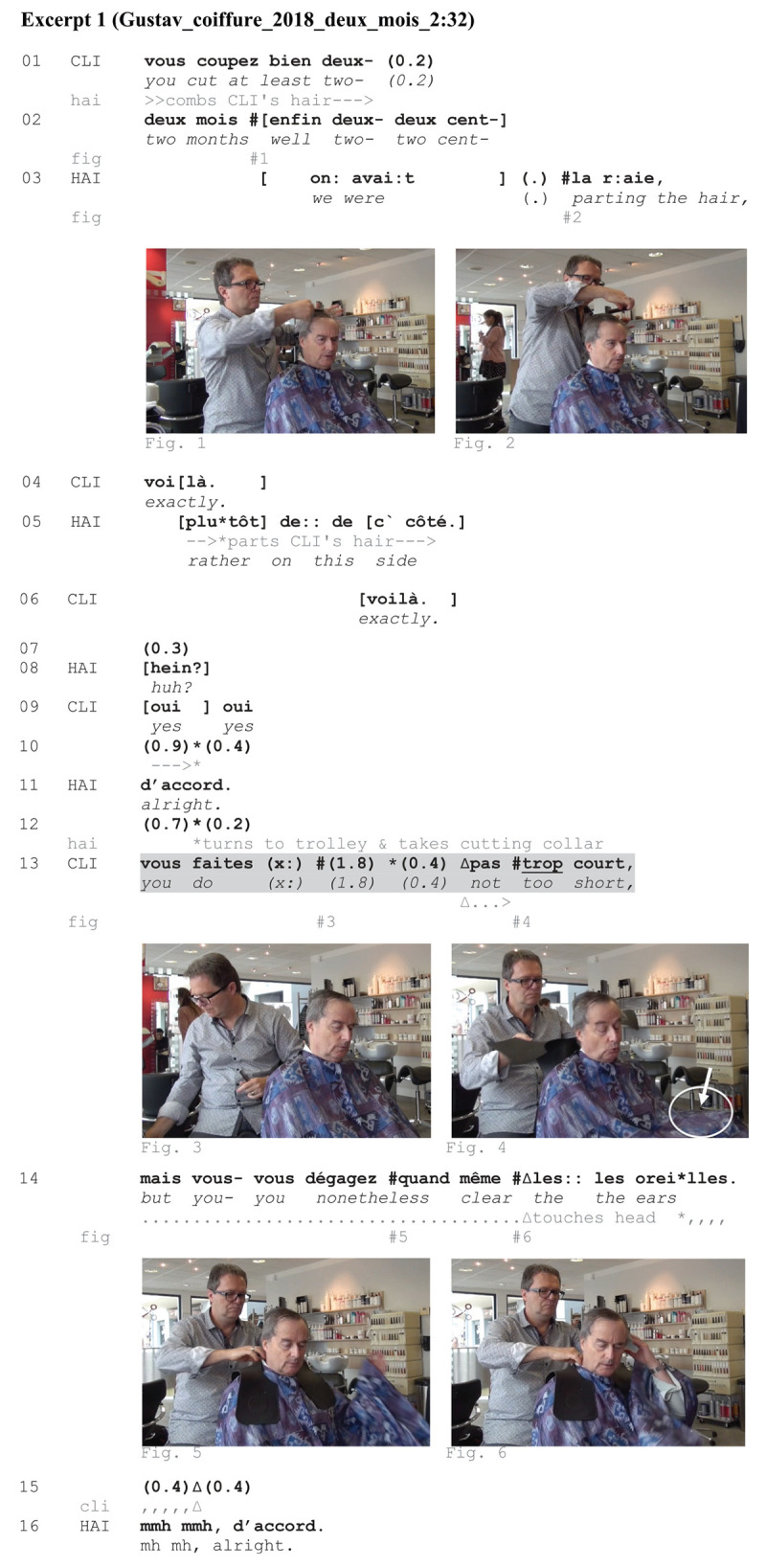


When the excerpt begins, CLI formulates an instruction (ll. 01-02), requiring HAI to remove the equivalence of 2 months of hair growth, which he tentatively reformulates in 2 cm (l. 02). While combing the hair (Figure 1), HAI intervenes in overlap by soliciting CLI’s approval about the correct parting of his hair, to which CLI responds positively (ll. 04, 06, 09). Parting the hair on the correct side (Figure 2) is an action with professional-practical relevance. HAI orients to the upcoming cutting activity by putting CLI’s hair in its right shape. In this part of the encounter, CLI uses talk to instruct HAI about the amount of hair to be cut; no manual resources are observable. It is noteworthy that HAI is already working on the head, which is not available for CLI’s manual access.

The sequence could be complete at line 11. HAI turns to the trolley (Figure 3) where he takes a cutting collar. The spatial placement of this object around CLI’s neck (Figure 4) further progresses the hairdressing activity toward cutting. At this moment, CLI elaborates on his preceding instruction, asking HAI not to cut too short^9^ (l. 13)—a description that he relativizes immediately by requesting a good trim around the ears (l. 14). CLI’s negative request relates to his prior instruction (ll. 01–02) and serves as retracting his own overstatement (Couper-Kuhlen and Thompson, 2005). The positive formulation afterward (l. 14) comes as a new instruction concerning a specific zone on the head (i.e., the ears). CLI starts to lift his hand from under the cape while formulating the negative instruction about the amount of hair to be cut (Figure 3 has been captured slightly after the start of CLI’s hand movement). CLI then touches his left side while delivering the positive instruction about the ears (the onset of CLI’s hand movement is simultaneous with the syllable *les:* ‘the,’ l. 14). We identify different components within CLI’s self-touching practice: It has a *deictic* component (Goodwin, 2014), in that it delimits a specific area, as well as an *iconic* component (McNeill, 1985), because it mimics the shape (Figure 6) of the expected outcome of his haircut (on *environmentally coupled gestures*, see Goodwin, 2007). Focusing on one or the other component would not end in different descriptions of CLI’s actions at lines 13 and 14: CLI makes the professional understand what to do with his hair by mobilizing different linguistic and embodied resources. This seems to confirm that gesture is a primary resource in the situated accomplishment of instructions (De Stefani and Gazin, 2014; Deppermann, 2018).

This excerpt has shown an example of CLI using a negative format during the consultation phase. CLI’s negative-formatted turn is delivered and treated as an instruction. It is sequentially embedded in a series of other instructions and tied to the larger activity of the consultation. In this moment, both participants actively build intersubjectivity by collaboratively delineating the haircut in prospect using talk and manual resources. In sum, the sequential placement of CLI’s negative-formatted turn toward the beginning of the encounter and its co-occurrence with specific manual practices within a broader instructional sequence argue in favor of a specific grammatical-sequential-praxeological-embodied package (on *grammar-body package*, see Kärkkäinen and Thompson, 2018).

Excerpt 2 also occurs toward the initial phase of the encounter, and more precisely, at the very beginning of the visit. HAI has just arrived behind CLI with his trolley and launches the beginning of the hairdressing action (l. 01). CLI needs to have her roots done. She says in overlap that she does not want an orange strand, probably accompanying her turn with manual resources.^10^ CLI is not satisfied with her last color service. In this case, her instruction is also delivering a strong criticism:

**EXCERPT 2:**
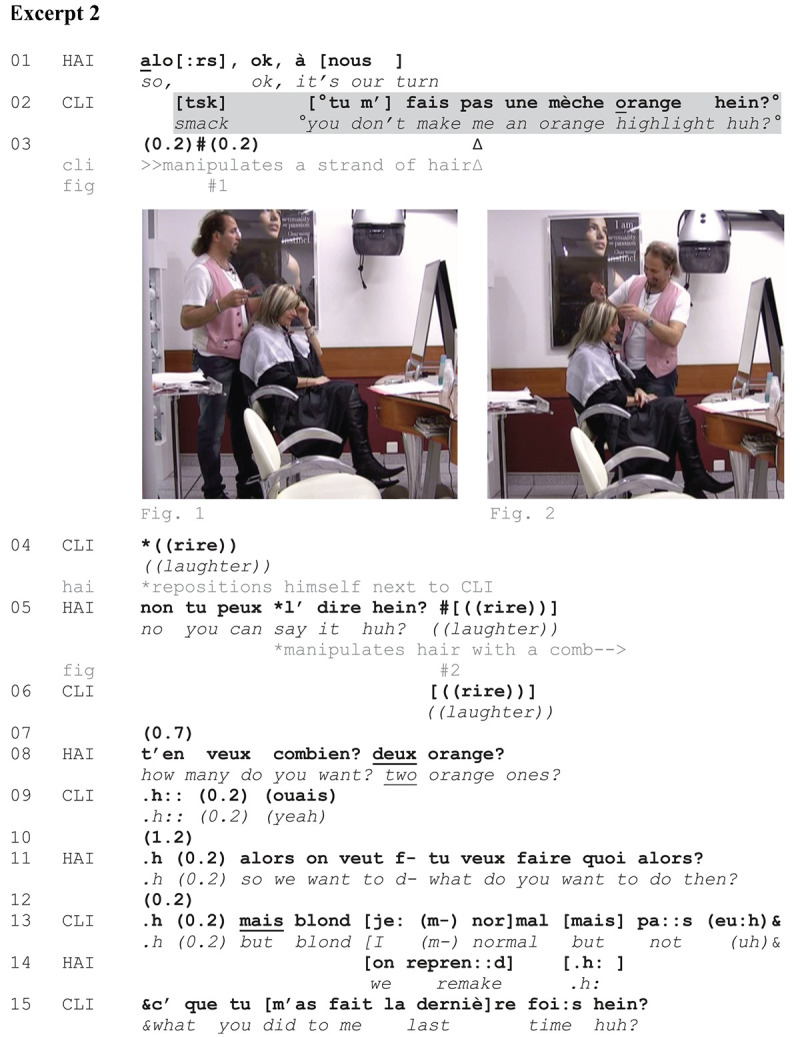


We understand from this excerpt that some of CLI’s strands after her last service went orange, whereas she likes them blond. CLI uses a negative format (l. 2) well before HAI’s referred-to action and possibly manipulates her hair simultaneously (see note 10). Captured slightly after her negative-formatted turn, Figure 1 shows that CLI seizes a strand of hair and stretches it, thereby maximizing its visibility and offering to HAI a visible account for her dissatisfaction (see Streeck’s, 2009, p. 49–57 notion of *dynamic grasp*). The fact that CLI initiates her request so early not only shows the familiarity between the two participants; CLI also displays that this is a follow-up visit, that is, her criticism is directly related to her previous visit. She exhibits that HAI has a personal responsibility in the matter. CLI’s turn is said in a lower voice, which could be oriented to the microphone, as if CLI wanted to report HAI’s fault to a third party (on *delicate formulations*, see Lerner, 2013). However, her laughter at line 04 allows CLI to mitigate her criticism “managing the socially delicate but institutionally required” (Raclaw and Ford, 2017, p. 1) voicing of a dissatisfaction. In the subsequent turn, HAI first takes the criticism upon himself (l. 05), but then laughs in overlap with CLI (Figure 2), showing that he does not orient to CLI’s turn as a serious matter. By so doing, he does not strongly disaffiliate with the criticism produced by CLI at line 02 (see Holt, 2012), but at the same time he aligns with CLI’s stance at line 04 by joining the laughter. At that moment, HAI has repositioned himself next to CLI (precisely on the side where she manipulated her hair) in a way that allows him to identify the problem (Figure 2). At line 08, HAI is even exaggerating by asking CLI how many orange strands she wants (l. 08), which shows some teasing going on. Concerning color issues, HAIs have the tendency not to treat the outcome of the coloring as their fault as professionals but as a possible result which is never entirely predictable (Horlacher, 2017). Teasing and laughing here open up the possibility that CLI was not entitled to do the criticism (at least, HAI downgrades his responsibility); laughing has also been described as doing some sort of relational work to remedy a previous transgression (Jefferson et al., 1987). At line 11, HAI is doing a re-beginning but CLI responds in relation to the previous sequence (l. 13). CLI is still focused on the color whereas HAI seems to initiate something else. CLI delivers the solicited instruction by expressing what she wants (*blond* and *normal*, l. 13), and what she does not want—with reference to her last visit. Again, the criticism is made obvious in her turn (ll. 13–15). CLI makes it clear that she does not want the same outcome as last time. She does not produce any further hair manipulations at that point, which can be accounted for by the fact that HAI has now diagnosed the problem and is working on the head.

To sum up: A close look at Excerpt 2 reveals that CLI’s negative-formatted turn does not exactly occupy the same sequential position as in Excerpt 1. CLI utters her turn at the very first occasion, while no words have been yet exchanged with HAI. This earliness might suggest that CLI’s turn is not a mere instruction in this case—an interpretation that is further supported by the prosodical shaping of her turn (and namely, CLI’s whispering voice) and HAI’s reaction.

We have seen so far that when coupled with a prevalent embodied conduct, namely hair manipulations, CLIs’ negative-formatted turns are presented and interpreted as instructions. During the consultation phase, hair manipulations from CLIs allow them to give instructions about specific areas or objects, pointing to them, delimiting them, or mimicking the shape of an expected outcome. Moreover, Excerpt 2 has shown that CLI’s turn is delivering a criticism. HAI’s reaction also supports this analysis: Unlike Excerpt 1, HAI treats CLI’s turn as laughable (Glenn, 2003) in Excerpt 2, and it might be precisely an appropriate response when avoiding criticism (see Holt, 2012 about laugh responses to defuse complaints).

In what follows (see section ‘Urgent Requests and Gaze Search’), CLIs can also be seen to challenge HAIs’ professional expertise through the use of negative requests. However, CLIs’ turns do not occur during the consultation but in a later phase of the encounter when HAI is already cutting or brushing CLI’s hair. In this case, they require an immediate response from HAI and are accompanied by different embodied conducts by the participants, namely CLI’s gaze search. CLI’s pursuit of mutual gaze with the professional seems to be seeking confirmation from HAI of the receipt of the directive and commitment of HAI to comply with the directive. Therefore, although CLIs’ grammatical formats look similar to Excerpts 1, 2, they do not constitute the same action. In Excerpts 3–5, CLIs’ turns are mostly delivered and interpreted as directives (and possibly as warnings, see ex. 5). This suggests that we can identify different interactional jobs that CLIs’ turns are brought to accomplish because of their different sequential environments, but also because of the participants’ different co-occurring embodied conducts.

### Urgent Requests and Gaze Search

In Excerpt 3, HAI has just switched on the hair-dryer and started to brush CLI’s hair by making a first curl. CLI closely monitors and challenges that action (l. 01).

**EXCERPT 3:**
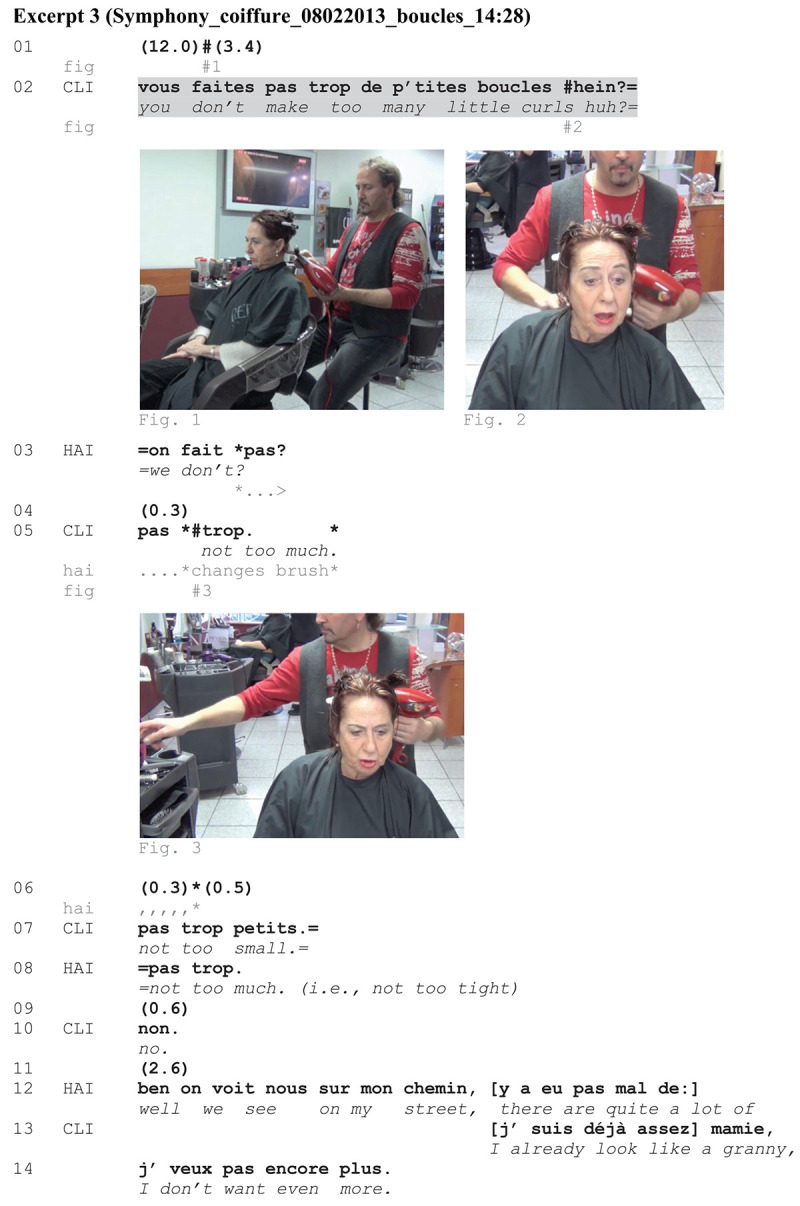


Through the same syntactic format described earlier, CLI demands that HAI not make too many little curls (l. 02). Near the end of her turn, CLI gazes at HAI in the mirror, raising her eyebrows, thereby actively soliciting a response (Figure 2). *On fait pas?* ‘we don’t?’ is a response to CLI’s turn by which HAI displays surprise and unexpectedness. As he was indeed making a little curl, HAI modifies the ongoing action as a response to CLI’s turn. By taking another brush on the trolley, he displays compliance with the action that is called for. His turn (l. 03) is at the same time treated by CLI as a first pair part initiating repair. A negotiation follows (ll. 05–10) in which CLI re-negotiates the quantification (l. 05) and the size of the curls (l. 07), as well as the degree of curliness (l. 08–10).

The sequence could be complete at line 11. The problem is solved, at least for HAI, who *links back* (De Stefani and Horlacher, 2008) to a discussion topic he has initiated before the sequence (on chatting in service encounters, see De Stefani and Horlacher, 2018; Horlacher, forthcoming). At that moment, CLI comes in overlap providing an account for why she prefers to not have too many curls: She associates little curls with grandmothers. Clients are entitled to their own opinions and choices about their appearance. Legitimate directives should not require further explanations. However, by accounting for her directive, CLI retrospectively softens her action and orients to being accountable for interfering in the implementation of the service, which is HAI’s field of expertise.

The analysis of Excerpt 3 has shown that CLI’s turn is a directive by means of which she imposes a change in the ongoing trajectory of action. The response-mobilizing potential of the negative format is enhanced by CLI’s embodied conduct soliciting a prompt reaction from HAI, in that case: The immediate suspension of the action that is called for. HAI immediately adjusts his professional practice in order to come to an agreement with CLI, thereby treating the requested action as legitimate.

Like Excerpt 3, Excerpt 4 will further illustrate that when CLI’s turn is a directive, it is associated with a distinct embodied conduct other than observed in Excerpts 1, 2. The excerpt occurs in the midst of a hair treatment. CLI demands HAI not to cut too much, while prompting a response by actively looking through the mirror, searching for HAI’s gaze (l. 02).

**EXCERPT 4:**
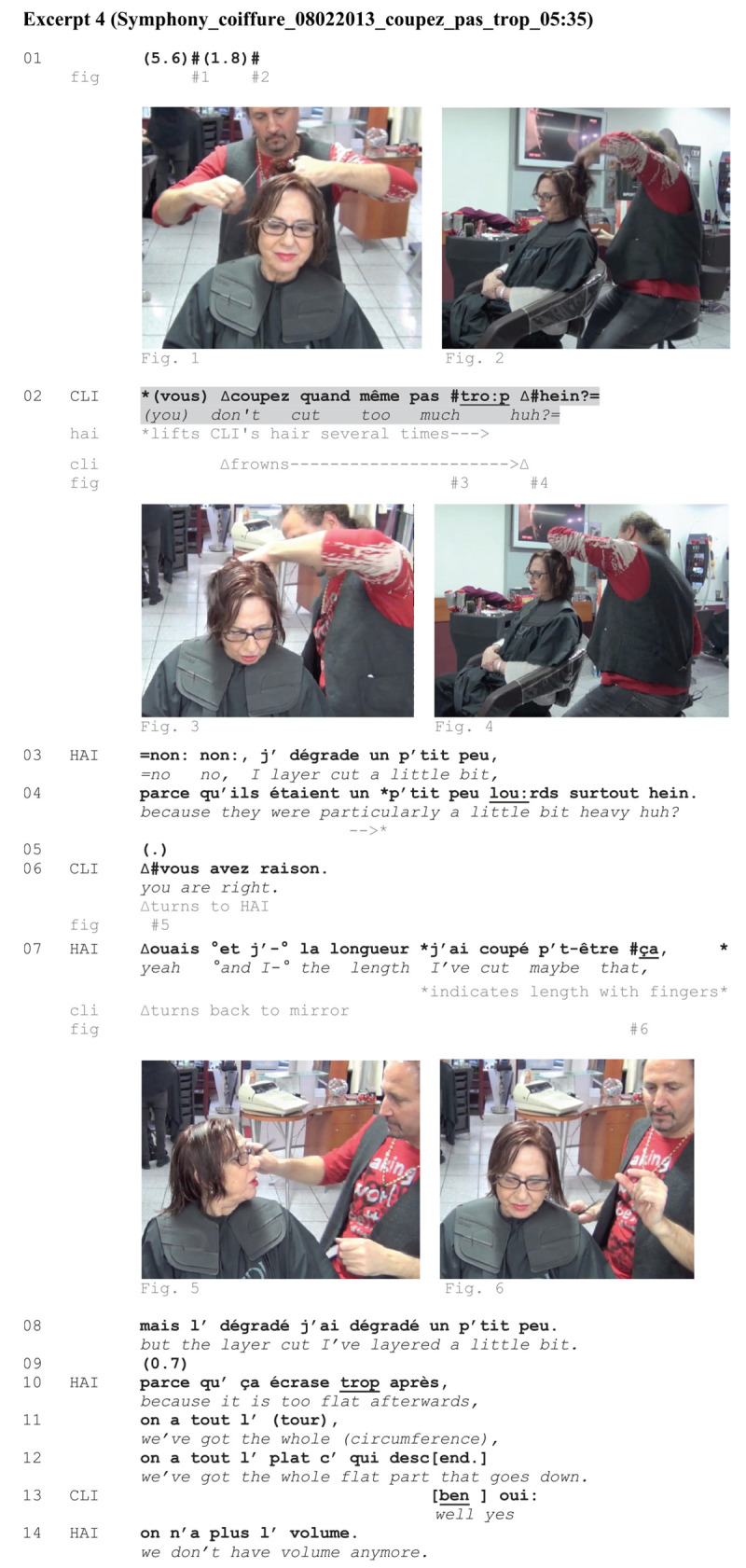


CLI demands that HAI not cut too much, mobilizing again a ‘you don’t do [action X] too much huh?’ format.^11^ Like in the preceding excerpt, CLI’s turn is associated with a typical embodied conduct: She slightly frowns (Figure 3) and stares through the mirror (Figure 4), trying to establish mutual gaze with HAI and to prompt a response. The professional delivers a ‘no’ in the subsequent turn, thereby showing that he treats CLI’s turn as a confirmation request. He then uses a specialized term replacing ‘to cut’ used by CLI by ‘to layer cut’ (l. 03). By using a different verb, HAI indicates that he is not doing the nominated action and CLI’s concern is unnecessary. He is done with the cutting and it is too late for CLI to negotiate or change the action trajectory. HAI further provides an account for his action (l. 04). At line 05, CLI provides a strong agreement, turning her head to HAI (Figure 5). She does not interact through the mirror anymore but establishes a reciprocal gaze with him. Through her embodied conduct, she thus continues to display what she had initiated at line 02, that is, the pursuit of a mutual gaze in a moment where intersubjectivity and trust are at stake. The sequence could be complete here but HAI keeps on giving explanations through lines 07 and 14. HAI provides an embodied representation of the length he has cut (Figure 6). This fits into a more general account that can possibly reassure CLI. By producing accounts (l. 04, ll. 10–14), HAI also displays his expertise concerning the hairdressing actions. He orients to being accountable for the service and works toward constructing shared understanding between the participants for a successful outcome of the haircut.

To sum up: What is CLI doing with her turn in line 02 in this excerpt? It first looks like a directive oriented to the suspension of HAI’s action. This interpretation is also supported by CLI’s embodied conduct. She frowns while uttering her negative-formatted turn and actively pursues a response from HAI, trying to establish mutual gaze through the mirror. From HAI’s perspective, however, it is impossible to comply with CLI’s demand because he is not cutting anymore. HAI treats CLI’s turn as a clarification request. However, it is noteworthy that HAI stops layer-cutting CLI’s hair at the back after this episode.

The last excerpt occurs at the end of a hair treatment. CLI had her hair brushed. The final step of her treatment consists in cutting the fringe. HAI has just seized a pair of scissors in his hand; it implies some risks. CLI demands HAI not to cut much, gazing insistently at him.

**EXCERPT 5:**
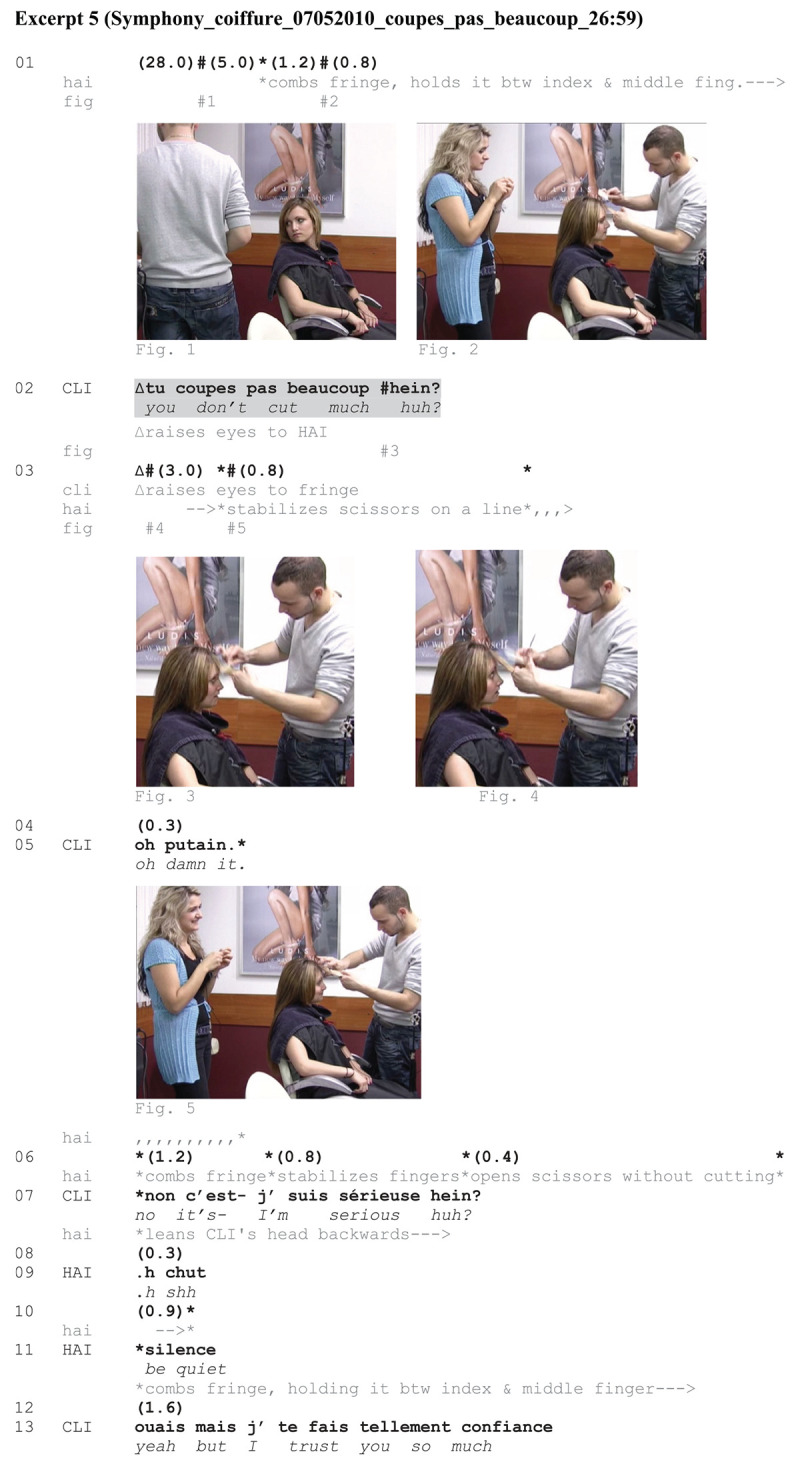


When the excerpt begins, CLI closely monitors HAI’s action (Figure 1). HAI has just taken scissors in his hand. He then combs CLI’s fringe, holding it between his index and middle finger (Figure 2). The action projected by HAI is imminent—as opposed to Excerpt 3 in which the action had just started, or to Excerpt 4 in which HAI’s action was under way (and even finished). Due to these temporal contingencies, CLI’s turn at line 02 in this excerpt could be interpreted as a warning, and CLI prefigures some blame if HAI fails to comply with it. Figure 3 shows that CLI is gazing at HAI when uttering her turn, thereby soliciting a response. However, HAI does not verbally answer. Instead, he indicates with the scissors a length to be cut which is certainly too short for CLI. Figure 4 shows that he is enacting precisely what CLI has said she does not want—as a joke. HAI embodies a possible non-compliance. CLI’s *oh putain* ‘oh damn it’ at line 05 treats HAI’s manual response as exaggerated and inappropriate. CLI’s turn was not a joke, as shown in line 07. However, HAI’s embodied response to CLI’s turn might also display a specific kind of relationship between the two participants. There is some teasing going on (see also ex. 2). It is excluded that HAI cuts the fringe that short. CLI’s *oh putain* ‘oh damn it’ while smiling (rather than a response to a genuine threat) shows exactly that. The apprentice who attends the scene is also smiling (Figure 5). At line 09, HAI’s response to CLI ‘sh be quiet,’ is so exaggerated that it is interpreted as a joke. At this point, CLI no longer negotiates on the forthcoming hairdressing action but lets HAI continue. If she did not trust HAI, she could have continued the negotiation on the matter. By producing the ironical *j’te fais tellement confiance* ‘I trust you so much’ (l. 13), CLI (jokingly) might be seen to account for her previous directive (‘you don’t cut much huh?’).

In this last excerpt, CLI gives her turn an urgent character, an analysis which is supported by her co-occurring embodied conduct, namely an insistent gaze search with HAI. CLI’s turn almost comes across like a warning. HAI takes into account CLI’s directive—not verbally but through an exaggerated gesture—teasing her. In other words, he produces an exaggerated gesture against the directive and later, a verbal joke, thus further teasing CLI.

To sum up: In the later phase of the encounter, CLI who produces the turn ‘you don’t do [action X] too much (huh)’ does not touch or lift her hands near her hair, which is now HAI’s working space. Instead, she gazes at HAI, soliciting a response, and seeking compliance. In Excerpts 3–5, CLI’s action is interpreted as a directive that directs HAI’s incipient or ongoing embodied action. As a response, HAI either modifies his ongoing embodied action or denies having acted in the nominated way, accounting for his ongoing embodied action.

## Conclusion

This paper set out to analyze clients’ use of a specific negative request format conjointly with the deployment of specific embodied resources in hair salons. The analysis has shown that when formatting their turns, clients can display by their co-occurring embodied conducts what type of action they are accomplishing.

Hair salons provide an original setting for the study of requests and allow for an innovative approach to service encounters. This study has taken into account the embodied realization of requests and investigated how the participants’ gaze, gestures, and manipulation of specific body parts are systemically implicated in the production and recognition of social actions. By focusing on the interplay between grammar and embodiment, we have highlighted the way a specific grammatical format ‘You don’t do [action X] too much (huh)’ is coupled with precise embodied resources for accomplishing particular actions: an instruction (sometimes tainted with criticism) or a directive (and possibly a warning). Focusing on where and how the clients’ negative-formatted turns are manifested and interpreted has delivered important insights on action formation and ascription in this type of encounter.

The main findings suggest that there is a tendency for the speakers to couple the ‘you don’t do [action X] too much (huh)’ with manual resources when accomplishing an instruction (ex. 1 and 2), whereas speakers use the same format as a directive with a different co-occurring embodied conduct: gaze search (ex. 3, 4, and 5). In the initial phase of the encounter when the participants negotiate on the forthcoming service, the client who produces the turn ‘you don’t do [action X] too much (huh)’ also reaches for his or her head and manipulates her or his own hair. In these cases, the client’s action is interpreted as an instruction concerning the hairdresser’s embodied future action. The hairdresser responds and displays his compliance with the instruction (or negotiates further on the topic) by using verbal means. In contrast, in the later phase of the encounter, when the professional is already engaged in the cutting or brushing activity, the client who produces the turn ‘you don’t do [action X] too much (huh)’ directs the hairdresser’s incipient or ongoing embodied action. In this second case, the clients’ turns are delivered and treated as urgent, precisely with regards to their timely position with relation to the hairdresser’s ongoing or projected embodied action. Urgent requests are thus deeply tied to the activities in which they are accomplished at a particular moment and clients self-select in response to a threat in the ongoing (or projected) professional’s hairdressing activity. As a response, the hairdresser modifies his ongoing embodied action or denies having acted in the nominated way. He can also respond with laughter or exaggerated gestures. However, it is very unlikely that the hairdresser does not comply with the client’s directive since it is obviously his interest that the client is happy with the outcome of the service. Hence, teasing and laughing might be appropriate responses when renegotiating expert-novice categories.

In sum, although the grammatical formats of the client’s turns are strikingly similar, the analysis has shown that the same syntactic format can be treated in very different ways. This shows how the formation/interpretation of social action is dependent on multiple factors: language (grammatical format) and the sequential environment in which it occurs with relation to the ongoing activity and embodied action. In doing so, the present article provides several empirical examples of the complexity of human action, and most specifically, adds to the understanding of human sociality as not limited to the use of language. Despite the currently increasing interest in the interrelation between language and participants’ embodied conduct, conversation analysis and interactional linguistics have not yet sufficiently dealt with this interplay in action formation. The present study about the distinct embodied conducts associated with later and urgent requests implies the need for other research in other settings, in order to further illustrate the distinction of how people do requesting in interaction.

## Data Availability Statement

The data analyzed in this study is subject to the following licenses/restrictions: The author does not have permission to share the video-recordings. Requests to access these datasets should be directed to the corresponding author.

## Ethics Statement

Ethical review and approval was not required for the study on human participants in accordance with the local legislation and institutional requirements. The patients/participants provided their written informed consent to participate in this study. Written informed consent was obtained from the individual(s) for the publication of any potentially identifiable images or data included in this article.

## Author Contributions

The author collected the main data corpus, carried out the empirical analyses, and authored the manuscript.

## Conflict of Interest

The author declares that the research was conducted in the absence of any commercial or financial relationships that could be construed as a potential conflict of interest.

## Publisher’s Note

All claims expressed in this article are solely those of the authors and do not necessarily represent those of their affiliated organizations, or those of the publisher, the editors and the reviewers. Any product that may be evaluated in this article, or claim that may be made by its manufacturer, is not guaranteed or endorsed by the publisher.

## Footnotes

1 We consider *requesting* as the overarching activity through which a participant solicits another participant. As for *requests*, they are the forms/the resources/the actions through which this activity is realized.

2 We use the action type ‘request’ as an umbrella term (‘umbrella action type’) and interpret the clients’ actions with the linguistic format ‘you don’t do [action X] too much (huh)’ as requests. We then go on dividing these ‘requests’ into ‘instructions’ and ‘directives.’ Not all CA researchers describe the action types ‘request,’ ‘instruction,’ and ‘directive’ in a similar way. Some scholars may use the action type ‘directive’ or ‘directive action’ as an umbrella term (Goodwin, 2006, p. 515; Rauniomaa, 2017, p. 326), but others may not. In his study on driving lessons, Deppermann (2018, p. 267) uses the umbrella term ‘instructions’ to describe actions produced by the instructor to advance the student’s learning process, and then distinguishes between instructions formulated as directives, requests, explanations, or descriptions.

3 Practices of touching have recently received increasing attention in different settings of interaction (see pioneering work by Cekaite, 2010; Nishizaka, 2011; Goodwin, 2017, but also Heath, 1986 on medical practitioners’ touching the patients’ bodies).

4 Turn-final *hein* is used a bit like a question tag, whereby speakers ask for confirmation of a previous assertion. Although it is not systematically accompanied with high rising intonation, this particle in French is strongly addressed to the interlocutor (Mondada, 2013a).

6 We have translated these turns in English with a simple present but the going-to-future or the simple future would maybe be more natural if we imagine a declarative syntax here.

7 It is a common belief that hairdressers always chop off too much. A quick visit to different hair forums and hair disaster websites supports this view of ‘scissor happy’ hairdressers.

8 The participants’ embodied conducts have been noted according to the transcription conventions developed by Mondada (latest version: 2019): https://www.lorenzamondada.net/multimodal-transcription.

9 In French, negation is formed with *ne* + conjugated verb + *pas*, while the particle *ne* is almost never pronounced in oral speech (Gadet, 1989). For this reason, it is unclear whether line 13 should be translated ‘you don’t do too short’ [negative form of the verb + too short] or ‘you do not too short’ [‘positive form of the verb + not too short’], depending if the negation affects the verb or the adjective. The 2.2 s pause in line 13 could argue for the first interpretation. Should we have a positive or negative formulation of the verb here, the whole turn remains a negatively formulated request.

10 Unfortunately, the first 5 s of the encounter have not been video recorded. They were captured by the audio-recorder placed on the table and by the wireless microphones but not by a camera. Since CLI is seen to be manipulating her hair right after uttering her negative-formatted turn (see Figure 1), we can reasonably infer that manual resources were already accompanying her action at line 02.

11 CLI’s turn possibly occurs in an imperative mode here (see section ‘Grammatical Features of Clients’ Requests’), whereas CLI uses the present tense in all other occurrences. There seems to be a correspondence between tense and specific temporal contingencies. Here, a more timely response is needed, since HAI’s action has been underway for quite a while. In Excerpts 1, 2, the nominated action has not yet started, whereas in Excerpt 3, CLI self-selects right after HAI makes a first curl.
